# Engineering cofactor supply and NADH-dependent d-galacturonic acid reductases for redox-balanced production of l-galactonate in *Saccharomyces cerevisiae*

**DOI:** 10.1038/s41598-020-75926-5

**Published:** 2020-11-04

**Authors:** Simon Harth, Jacqueline Wagner, Tamina Sens, Jun-yong Choe, J. Philipp Benz, Dirk Weuster-Botz, Mislav Oreb

**Affiliations:** 1 Faculty of Biological Sciences, Institute of Molecular Biosciences, Goethe University Frankfurt, Max-von-Laue Straße 9, 60438 Frankfurt am Main, Germany; 2 Institute of Biochemical Engineering, Technical University of Munich, Boltzmannstr. 15, 85748 Garching, Germany; 3 Department of Chemistry, East Carolina Diabetes and Obesity Institute, East Carolina University, 115 Heart Drive, Greenville, NC 27834 USA; 4 Department of Biochemistry and Molecular Biology, Rosalind Franklin University of Medicine and Science, 3333 Green Bay Road, North Chicago, IL 60064 USA; 5 TUM School of Life Sciences Weihenstephan, Holzforschung München, Technical University of Munich, Hans-Carl-von-Carlowitz-Platz 2, 85354 Freising, Germany

**Keywords:** Metabolic engineering, Oxidoreductases

## Abstract

d-Galacturonic acid (GalA) is the major constituent of pectin-rich biomass, an abundant and underutilized agricultural byproduct. By one reductive step catalyzed by GalA reductases, GalA is converted to the polyhydroxy acid l-galactonate (GalOA), the first intermediate of the fungal GalA catabolic pathway, which also has interesting properties for potential applications as an additive to nutrients and cosmetics. Previous attempts to establish the production of GalOA or the full GalA catabolic pathway in *Saccharomyces cerevisiae* proved challenging, presumably due to the inefficient supply of NADPH, the preferred cofactor of GalA reductases. Here, we tested this hypothesis by coupling the reduction of GalA to the oxidation of the sugar alcohol sorbitol that has a higher reduction state compared to glucose and thereby yields the necessary redox cofactors. By choosing a suitable sorbitol dehydrogenase, we designed yeast strains in which the sorbitol metabolism yields a “surplus” of either NADPH or NADH. By biotransformation experiments in controlled bioreactors, we demonstrate a nearly complete conversion of consumed GalA into GalOA and a highly efficient utilization of the co-substrate sorbitol in providing NADPH. Furthermore, we performed structure-guided mutagenesis of GalA reductases to change their cofactor preference from NADPH towards NADH and demonstrated their functionality by the production of GalOA in combination with the NADH-yielding sorbitol metabolism. Moreover, the engineered enzymes enabled a doubling of GalOA yields when glucose was used as a co-substrate. This significantly expands the possibilities for metabolic engineering of GalOA production and valorization of pectin-rich biomass in general.

## Introduction

The polysaccharide pectin, a constituent of all plant cell walls, is abundantly present in agricultural residues of many commodity crops, exceeding production rates of several hundred million tons each year^1,2^. However, despite holding great promise as a source of platform chemicals, it is currently underutilized, serving mainly as a gellant or low-cost animal feed and a significant fraction is only being burned. With a mass content of about 70%, d-galacturonic acid (GalA) is the major backbone sugar of pectin^2^. Due to its high abundance, many microbial species can utilize GalA as a carbon source through different pathways^1,3–5^, while *Saccharomyces cerevisiae*, as one of the most popular microbial chassis organisms, does not have this ability. Several groups have attempted to transfer GalA utilization genes from bacteria or fungi into *S. cerevisiae*. Whereas the establishment of a fully functional bacterial (isomerase) pathway in yeast proved challenging^6^, likely due to the involvement of enzymes dependent on iron-sulfur clusters that are notoriously difficult to express in the eukaryotic host, fungal enzymes could be successfully expressed^7–10^. Fungal GalA catabolic pathways, as first described in the mold *Trichoderma reesei* (*Hypochrea jecorina*)^11,12^, are functionally conserved^13^. As a first step, they involve GalA reductases (EC 1.1.1.365), which convert GalA to l-galactonate (GalOA) using NADPH as a reducing equivalent. GalOA is subsequently converted to 2-keto-l-galactonate by an l-galactonate dehydratase (EC 4.2.1.146). In the next step, the C6-backbone is cleaved by a 2-keto-3-deoxy-galactonate aldolase (EC 4.1.2.54) into pyruvate and l-glyceraldehyde. The latter is finally reduced to glycerol by l-glyceraldehyde reductase (EC 1.1.1.372), which is the second NADPH-dependent enzyme of this pathway. Glycerol and pyruvate are common metabolic intermediates in fungi providing both energy and carbon skeletons. Thus, GalA can serve as a sole carbon source in fungi^13^.

Notably, GalOA is not only the first intermediate of the fungal GalA catabolism but also, as a polyhydroxy acid, a compound of potential industrial interest^14,15^. Related compounds, such as gluconate (E574), have a long history of industrial use with applications in different areas of cosmetics, food and beverage industry, e.g. as complexing agents or acidifiers. Also, l-galactono-γ-lactone (GgL), the product of spontaneous lactonization of GalOA, is an analog to glucono-δ-lactone (E575), a common slow-release acidifier in food. Last, but not least, GalOA is a precursor in the biotechnological production of l-ascorbic acid (vitamin C) via a further enzymatic step after spontaneous lactonization to GgL^16,17^.

Based on the above-mentioned potentials, we targeted the optimized production of GalOA (and GgL as a spontaneously formed byproduct) in *S. cerevisiae*. This requires the expression of a heterologous GalA reductase and a sufficient supply of redox equivalents to drive the reduction. The first GalA reductase was identified in *Hypocrea jecorina* (TrGar1) and was shown to be NADPH-specific^11^. The later identified Gar1 enzymes from *Cryptococcus diffluens*^9^ and *Rhodosporidium toruloides*^13^ are phylogenetically related to TrGar1. Interestingly, although the mold *Aspergillus niger* also has a TrGar1 homolog (AnGar1; encoded by gene An16g04770; UniProt Acc. Number A2R7U3), another protein named AnGaaA (encoded by An02g07710) was identified as the *bona fide* GalA reductase in this organism^18^. AnGaaA is not related to Gar1 proteins and accepts NADPH as well as NADH as cofactor. However, its catalytic efficiency (measured as k_cat_/K_m_ ratio) for GalA reduction is approximately 50-fold higher with NADPH than with NADH^18^. Previous studies reported that the production of GalOA via GalA reductases^9^ or utilization of GalA as a carbon source through an introduced fungal pathway^7,8^ in *S. cerevisiae* was only possible in the presence of a co-substrate such as the hexoses glucose, galactose and fructose^7,9^ or the pentoses xylose and arabinose^8^. This is obviously necessary due to the high oxidation state of GalA, whose reduction to GalOA requires the input of NADPH derived from the metabolism of co-substrates. Notably, a systematically optimized expression of a fully functional fungal catabolic pathway led to utilization of GalA as a sole carbon source by *S. cerevisiae*^10^, which could be strongly improved by concomitantly enhancing the catabolism of glycerol, one of the intermediates resulting from GalA metabolism (see above). This observation is consistent with the notion that fungi with the natural ability to grow on GalA as a sole carbon source appear to have also a strong glycerol catabolism, which yields the necessary reducing equivalents^13^. Interestingly, AnGaaA expression conferred a more robust growth of *S. cerevisiae* on GalA than TrGar1, despite a higher specific activity of the latter enzyme. This was attributed to the ability of AnGaaA to utilize NADH^10^. Based on these notions, we reasoned that the supply of suitable redox cofactors is a crucial engineering target for GalOA production and GalA utilization as a carbon source in *S. cerevisiae*. To test the concept, we first developed a co-fermentation system utilizing the sugar alcohol sorbitol as a redox donor to produce a “surplus” of NADH or NADPH necessary for GalA reduction. In this system, the GalA reductases could be conveniently assayed for their cofactor specificity. The biocatalytic process performance of selected strains was studied in batch processes making use of fully controlled stirred-tank bioreactors. Using structure-guided mutagenesis, we identified mutations in AnGar1 that enable the enzyme to use either NADPH and NADH or NADH exclusively. We demonstrate that higher yields of GalOA per consumed glucose can be produced with the mutant enzyme variants compared to their wildtype counterparts. This work thereby expands the toolbox necessary for valorization of complex carbohydrate mixtures present in hydrolysates of pectin-rich biomass^19^.

## Results and discussion

### Establishing a system for redox-balanced GalA reduction

**Figure 1:**
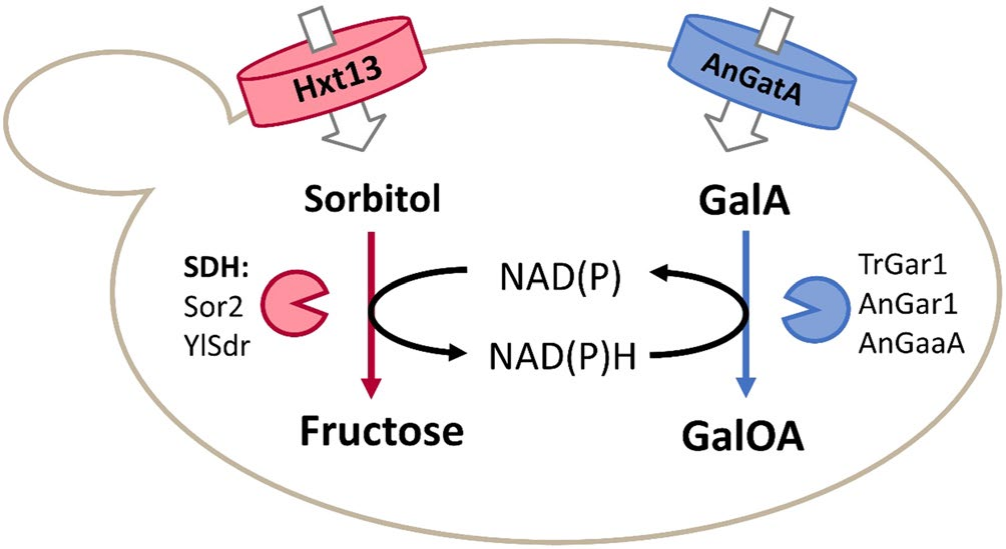
Redox-balanced system for GalOA production. The cofactors necessary for the reduction of d-galacturonate by GalA reductases (TrGar1, AnGar1 or AnGaaA) to GalOA can be derived from the oxidation of sorbitol by SDH. By choosing the suitable SDH, either NADH (Sor2) or NADPH (YlSdr) can be accumulated. Fructose produced by SDH subsequently enters glycolysis.

Previous work by others has shown that, due to its high oxidation state, the reduction of GalA can occur only in the presence of a co-substrate (redox-donor) such as fructose or glucose, but the molar yield of GalOA per mol co-substrate was low^9^. Presumably, the low yields were due to insufficient redox power provided by hexose metabolism. Therefore, we decided to couple the reduction of GalA to the oxidation of the sugar alcohol sorbitol, which exhibits a higher reduction state compared to glucose. Sorbitol must be oxidized to fructose by a sorbitol dehydrogenase (SDH) to enter glycolysis. Thereby, SDH can provide the necessary reducing equivalents in a stoichiometric manner (Fig. 1).

For co-utilization of sorbitol and GalA, different genetic cassettes, each for overexpression of four genes—a sorbitol transporter, an SDH, a GalA transporter and a GalA reductase—were constructed and integrated into the *URA3* locus of the hexose-transporter deficient (*hxt*^*0*^) strain EBY.VW4000^20^. This strain background was chosen to rule out any influence of endogenous hexose transporters on GalA uptake^10^. All cassettes contained the endogenous transporter *HXT13* for sorbitol uptake^21^ and GatA from *A. niger*^10^ for GalA uptake. As GalA reductases, we chose the well-known enzyme TrGar1 and its orthologue from *A. niger* (AnGar1), for which a GalA reductase activity had not been demonstrated yet. To provide NADPH or NADH, we selected the YlSdr from *Yarrowia lipolytica*^22^ or the endogenous Sor2^21^, respectively. The different combinatorial cassette configurations and resulting strains are listed in Table 1.

**Table 1:** List of yeast strains used in this study. For the genotypes, the standard nomenclature is used. Under “relevant genotype” the parental strains are indicated in bold. The open reading frames relevant for GalA utilization are underlined. The prefixes “p” and “t” denote promoters and terminators, respectively.

Strain name	Relevant genotype	Source
CEN.PK2-1C	*MATa leu2-3,112 ura3-52 trp1-289 his3-1 MAL2-8c SUC2*	EUROSCARF
EBY.VW4000	**CEN.PK2-1C** *Δhxt1-17 Δgal2 Δstl1::loxP Δagt1::loxP Δmph2::loxP Δmph3::loxP*	Reference^20^
JWY019	*MATa MAL2-8c SUC2 Δilv2 Δbdh1 Δbdh2 Δleu4 Δleu9 Δecm31 Δilv1 Δadh1 Δgpd1 Δgpd2*	Reference^30^
SiHY001	**EBY.VW4000** *Δura3::pCCW12-AnGATA-tPGK1-pPGK1-AnGAR1-tENO1-pTDH3-HXT13-tSSA1-pTEF2-YlSDR-tADH1-pAgTEF-KanMX-tAgTEF;* constructed from transformation with SiHV040	This study
SiHY002	**EBY.VW4000** *Δura3::pCCW12-AnGATA-tPGK1-pPGK1-TrGAR1-tENO1-pTDH3-HXT13-tSSA1-pTEF2-YlSDR-tADH1- pAgTEF-KanMX-tAgTEF;* constructed from transformation with SiHV041	This study
SiHY003	**EBY.VW4000** *Δura3::pCCW12-AnGATA-tPGK1-pPGK1-AnGAR1-tENO1-pTDH3-HXT13-tSSA1-pTEF2-SOR2-tADH1- pAgTEF-KanMX-tAgTEF;* constructed from transformation with SiHV042	This study
SiHY004	**EBY.VW4000** *Δura3::pCCW12-AnGATA-tPGK1-pPGK1-TrGAR1-tENO1-pTDH3-HXT13-tSSA1-pTEF2-SOR2-tADH1- pAgTEF-KanMX-tAgTEF;* constructed from transformation with SiHV043	This study
SiHY007	**EBY.VW4000** *Δura3::pCCW12-AnGATA-tPGK1-pTDH3-HXT13-tSSA1-pTEF2-YlSDR-tADH1-pAgTEF-KanMX-tAgTEF;* constructed from transformation with SiHV046	This study
SiHY008	**EBY.VW4000** *Δura3::pCCW12-AnGATA-tPGK1-pTDH3-HXT13-tSSA1-pTEF2-SOR2-tADH1-pAgTEF-KanMX-tAgTEF;* constructed from transformation with SiHV047	This study
SiHY030	**CEN.PK2-1C** *Δura3::pCCW12-AnGATA-pPGK1-AnGAR1 [R267L]-pTDH3-HXT13-pTEF2-SOR2-KanMX-tAgTEF;* constructed from transformation with SiHV129	This study
SiHY032	**CEN.PK2-1C** *Δura3::pCCW12-AnGATA-pPGK1-AnGAR1 [K261M, R267L]-pTDH3-HXT13-pTEF2-SOR2-KanMX-tAgTEF;* constructed from transformation with SiHV128	This study
SiHY062	**JWY019** *Δura3::pCCW12-AnGATA-tPGK1-pPGK1-AnGAR1 [R267L]-pAgTEF-KanMX-tAgTEF;* constructed from transformation with SiHV136	This study
SiHY063	**JWY019** *Δura3::pCCW12-AnGATA-tPGK1-pPGK1-AnGAR1 [R267L, K261M]-pAgTEF-KanMX-tAgTEF;* constructed from transformation with SiHV137	This study
SiHY072	**JWY019** *Δura3::pCCW12-AnGATA-tPGK1-pPGK1-AnGAR1-pAgTEF-KanMX-tAgTEF;* constructed from transformation with SiHV158	This study

**Figure 2:**
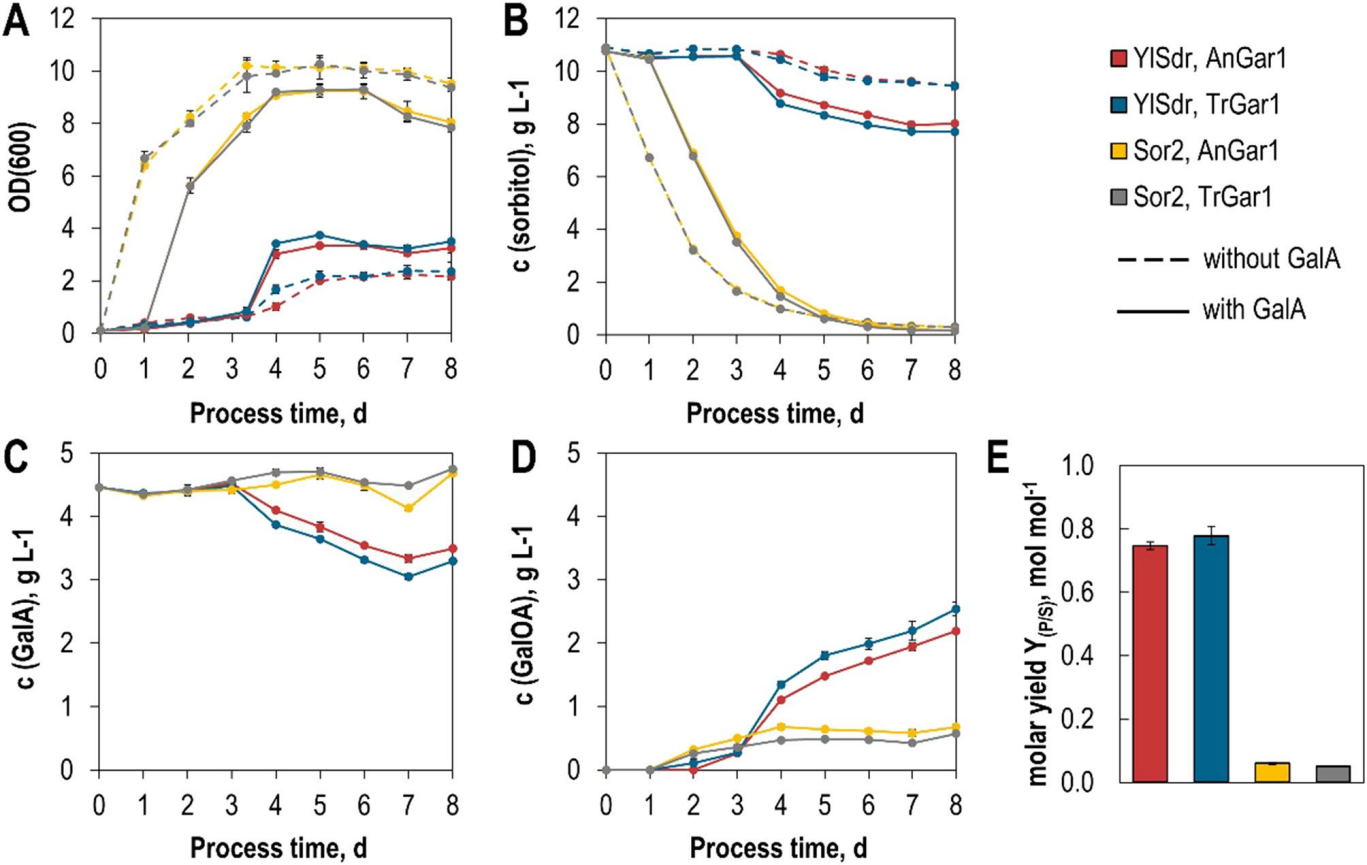
Co-fermentation of GalA and sorbitol. Yeast strains SiHY001-004 expressing indicated enzyme combinations were cultivated in shake flasks in phosphate-buffered SC-media with sorbitol as carbon source either without (dashed lines) or with (solid lines) GalA. Cell growth was monitored photometrically (OD_600_). Concentrations of sorbitol, GalA, and GalOA were measured by HPLC. Mean values and standard deviations of biological triplicates are shown. Error bars may be smaller than the symbols. Molar yields (Y_P/S_) were calculated as mol GalOA produced per mol of sorbitol consumed after 8 days of cultivation. The same color code is applied in all panels.

To test the concept, we performed shake flask fermentations under aerobic conditions with these strains in sorbitol-containing media with or without GalA supplementation (Fig. 2).

In line with previous observations^21^, the (NAD^+^-dependent) Sor2 conferred robust growth and sorbitol consumption, both of which were markedly delayed by the addition of GalA. This is likely due to the weak acid toxicity and/or inhibition of sorbitol uptake, as Hxt13 is closely related to the yeast galactose permease Gal2 that was previously reported to be competitively inhibited by GalA^23^. With Sor2, only low concentrations of GalOA (up to 0.68 g L^−1^) were produced. The behavior of strains expressing the NADP^+^-dependent YlSdr was the opposite. Without GalA, only marginal growth and a concomitantly slow sorbitol consumption were measured. This can be explained by the accumulation of cytosolic NADPH that, in contrast to NADH, cannot be re-oxidized, leading to a growth arrest^24^. As expected, the addition of GalA stimulated growth and sorbitol consumption by acting as a redox sink for the accumulating NADPH (see Fig. 1). This is reflected by the substantially higher titer of GalOA in fermentations with YlSdr (up to 2.54 g L^−1^). For both GalA reductases, TrGar1 and AnGar1, resulting titers and molar yields per mol consumed co-substrate (Y_P/S_) were similar. Despite slower growth and slower sorbitol consumption, these yields were about 12-fold higher with NADPH- than NADH-dependent SDH. Altogether, these results show that the supply of suitable reducing equivalents is the most critical engineering target for GalOA production. Moreover, we demonstrate, for the first time, the activity of AnGar1 as a GalA reductase.

**Figure 3:**
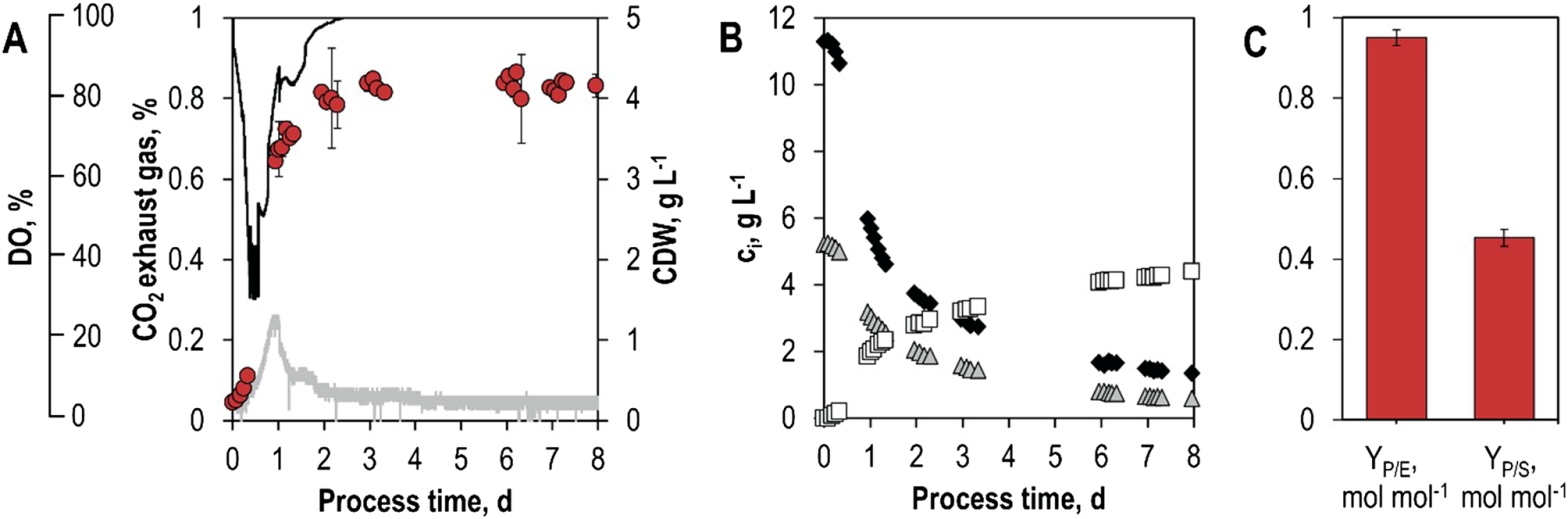
Aerobic batch fermentation of *S. cerevisiae* SiHY001 in a stirred-tank bioreactor. Concentration of cell dry weight (circles), online measured dissolved oxygen (DO) concentration (black line), and carbon dioxide (CO_2_) concentration (grey line) in the exhaust gas as a function of the process time (**A**). Concentrations of sorbitol (diamonds), GalA (triangles), and GalOA (rectangles) as a function of the process time, as measured via HPLC in the fermentation broth (**B**). Final molar yield coefficients Y_P/E_ (GalOA/GalA) and Y_P/S_ (GalOA/sorbitol) (**C**). Error bars represent the standard deviation of two individual batch experiments in stirred-tank bioreactors.

Growth, sorbitol and GalA consumption, as well as GalOA formation of engineered *S. cerevisiae* were also studied in aerobic batch-operated, fully controlled stirred-tank bioreactors (e.g., pH, DO, power input). Figure 3 shows online and offline monitored state variables of the standardized batch process with the best performing strain of shake flask experiments (*S. cerevisiae* SiHY001 expressing YlSdr and AnGar1). By using pre-cultures grown in the same medium as for batch processes, the lag phase observed in shake flask experiments could be substantially reduced to a few hours. The highest metabolic activity was measured within the first 24 h of the batch process, reaching a CO_2_ concentration of 0.25% (v/v) in the exhaust gas and a maximum growth rate of 0.12 h^−1^. Finally, 0.45 mol GalOA was formed per mol sorbitol consumed (Y_P/S_), indicating that more than 50% of sorbitol was used for CO_2_ and biomass formation. Growth and sorbitol consumption rates started to decrease at a process time of 24 h, despite the high remaining sorbitol concentration in the reactor (5.7 g L^−1^/31.3 mM). The decrease in the sorbitol consumption rate may be caused by the rather low affinity (K_M_ = 20.4 mM) of Hxt13 for sorbitol^21^ and/or accumulation of the possibly toxic product GalOA. Even without further growth of the *S. cerevisiae* cells beyond 72 h, GalOA was still formed until a final concentration of 4.4 g L^−1^ after a process time of 191 h. During the stationary phase (72–191 h), the yield coefficient for GalOA on sorbitol (Y_P/S, 72–191 h_) increased to 0.79 mol mol^−1^, which is comparable to the results observed in shake flasks (Fig. 2) where the growth of the yeast cells was reduced considerably compared to that in the stirred-tank bioreactor. The substrate-specific product selectivity (Y_P/E_) of 0.95 mol GalOA per mol GalA (0.93 g GalOA g^−1^ GalA) indicates a nearly by-product free biotransformation and exceeds selectivities of filamentous fungi under similar conditions in batch processes (0.6–0.9 g GalOA g^−1^ GalA^15^). The carbon balance of the batch process could be closed (100% considering an error range of ± 10%) (Supplementary Fig. S3).

### Engineering GalA reductases for NADH-specificity

**Figure 4:**
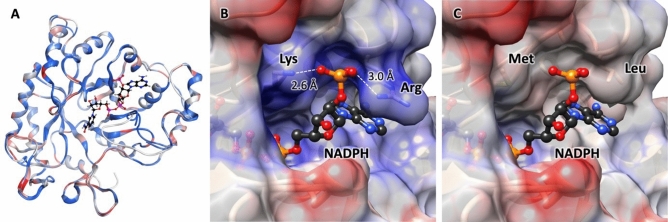
Structural homology models of TrGar1 and AnGar1. The homology structural models of TrGar1 and AnGar1 were based on the crystal structure of the NADPH-dependent aldehyde reductase AKR1A1 (PDB ID 1HQT). (**A**) Overlay of the structures of TrGar1 and AnGar1 showing the binding site of NADPH. The coloring reflects the degree of residue conservation: dark blue indicates identical residues, with light blue are similar residues, in red are non-conserved substitutions in TrGar1 compared with AnGar1. (**B**) and (**C**) Close-up of the binding site for the phosphoryl moiety of NADPH in the wild-type (**B**) vs. the double mutant (**C**) enzymes (TrGar1 K254M/R260L or ArGar1 K261M/R267L). The double mutant lacks the electrostatic interactions of the NADPH phosphoryl group with Lys (254 in TrGar1 or 261 in ArGar1) and Arg (260 in TrGar1 or 267 in ArGar1) residues. The coulombic protein surface calculated with UCSF Chimera (www.rbvi.ucsf.edu/chimera) shows positive charge in blue and negative charge in red. NADPH is shown as ball-and-stick model.

To identify the amino acid residues responsible for NADPH binding, we generated the structural models of TrGar1 and AnGar1. The crystal structure of the NADPH-dependent aldehyde reductase AKR1A1 from *Sus scrofa* (PDB ID 1HQT) was the template; this enzyme shares 37% sequence identity with TrGar1 and AnGar1. The homology models of TrGar1 and AnGar1 were constructed in Molecular Operating Environment (MOE; Chemical Computing Group, https://www.chemcomp.com/). Given the high sequence homology between TrGar1 and AnGar1 (63% identity and 81% similarity), their structural models are quite similar (Fig. 4A). In the active site, the phosphoryl group of NADPH interacts electrostatically with the two positively charged residues Lys (254 in TrGar1 or 261 in AnGar1) and Arg (261 in TrGar1 or 267 in AnGar1) (Fig. 4B). In MOE, we performed mutation scanning of these residues and examined the protein stability as well as NADH versus NADPH ligand affinity for the various substitutions (Supplementary Fig. S4).

According to the protein stability results, the lysine residue could be mutated to Leu, Met, Phe, Trp, or Tyr; among these, the mutant to Met also has a better affinity for NADH than for NADPH, which is predictable given that Met is similar in size to Lys, but without the positive charge (Supplementary Fig. S4). A similar analysis for the arginine residue indicates that, while Ile, Leu, Thr, and Val are stable alternatives for its mutagenesis, the substitution with Leu offers better protein stability and ligand affinity with NADH (Supplementary Fig. S4). Furthermore, both K261M and R267L preserve the space occupied by Lys and Arg, respectively, in the NADP binding site well. Therefore, we hypothesized that the amino acid substitutions K254M and R260L in TrGar1 or the corresponding mutations in AnGar1 (K261M and R267L, respectively), alone or in combination, will result in the desired change of cofactor-specificity (Fig. 4C).

The ability of GalA reductase variants to utilize different cofactors was tested using the sorbitol co-fermentation system as described above, with the exception that enzymes were expressed from multicopy (2µ) plasmids. AnGaaA, a phylogenetically non-related GalA reductase, which naturally accepts NADPH and, albeit to a lesser extent, NADH^18^ was included for comparison.

**Figure 5:**
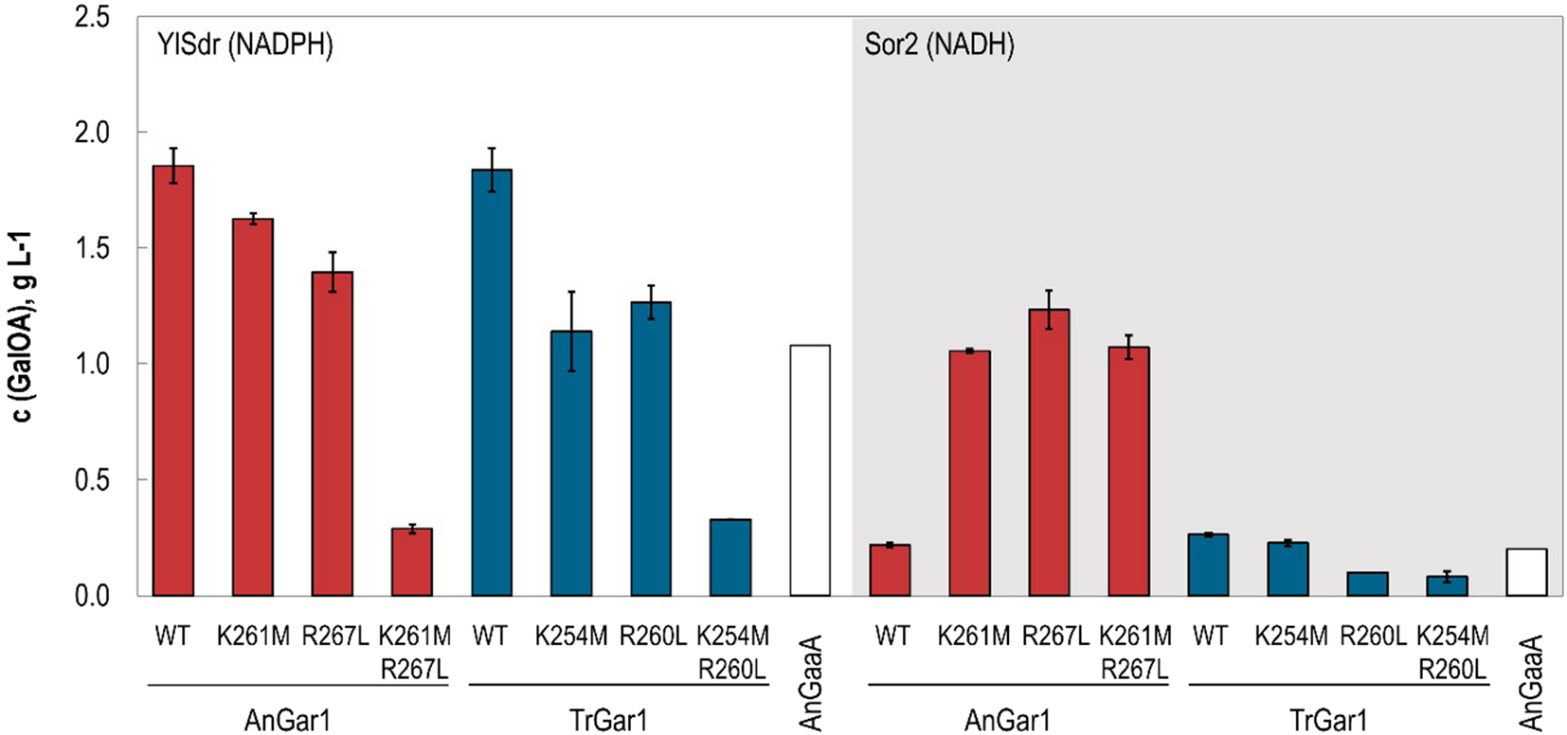
Cofactor specificity of GalA reductase variants. Indicated GalA reductase variants were overexpressed from multicopy plasmids in the strains SiHY007 (YlSdr) and SiHY008 (Sor2) together with NADPH- or NADH-dependent SDH YlSdr or Sor2, respectively. The conversion of GalA into GalOA was measured in culture supernatants of shake flasks after 7 days of cultivation by HPLC analysis. AnGaaA, which naturally accepts NADPH and also NADH, was included for comparison. Mean values and standard deviations of biological triplicates are shown.

As expected, the two wildtype GalA reductases could only produce a considerable amount of GalOA when in combination with YlSdr, i.e., with NADPH as a cofactor (Fig. 5). The lower titers observed with AnGaaA in comparison to TrGar1 correlate with specific activity differences of these enzymes in yeast, as previously reported^10^. Wildtype TrGar1 and AnGar1 produce comparable amount of GalOA. The mutated variants of TrGar1 showed a decreased GalOA production with NADPH, as expected, but no considerable product formation in the NADH background. In contrast, both single amino acid substitutions K261M and R267L conferred AnGar1 the ability to accept NADH in addition to NADPH. The double mutant K261M/R267L apparently confers NADH-specificity to AnGar1, since high amounts of GalOA are only produced in combination with Sor2. Importantly, the mutated AnGar1 variants are superior to AnGaaA with NADH, demonstrating the feasibility of the enzyme engineering approach.

**Figure 6:**
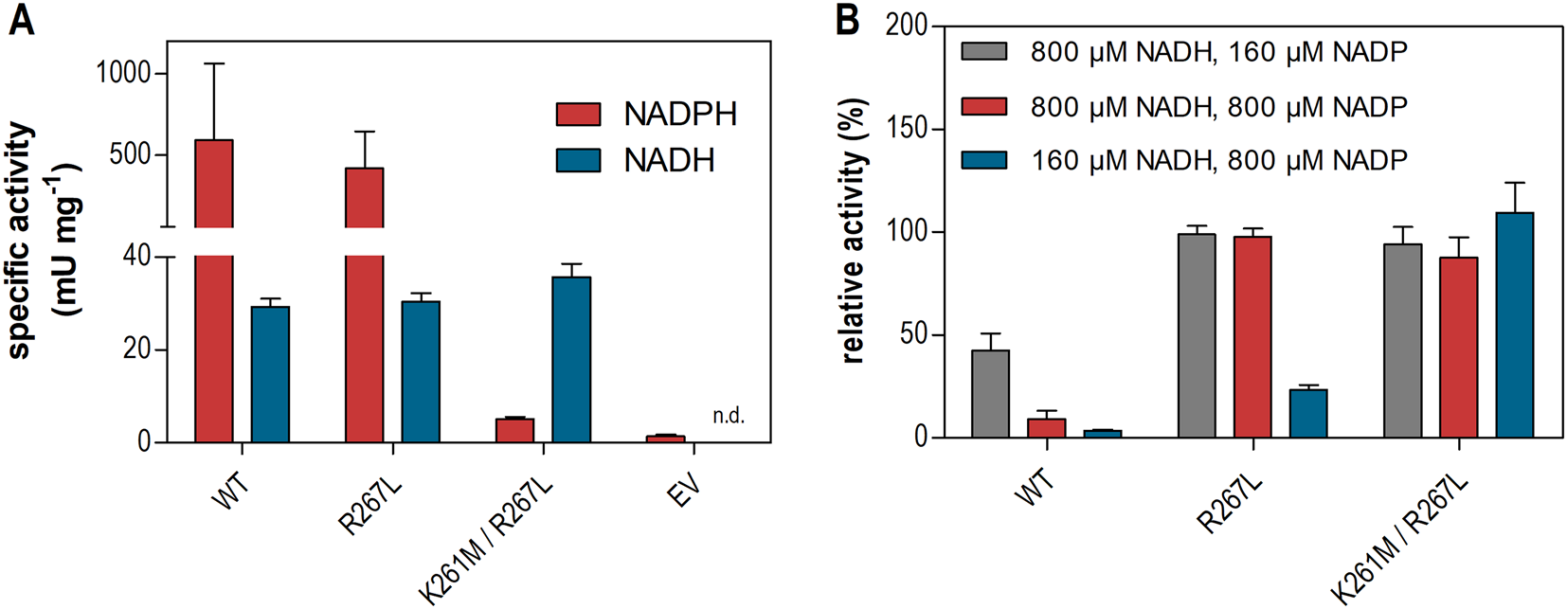
Enzyme activity assay of AnGar1 variants. The enzymes were expressed from plasmids in CEN.PK2-1C cells. The cells transformed with the empty vector (EV) were used as a negative control. In (**A**), the assays were performed with NADPH or NADH alone. The specific activity (mili Units per mg protein, mU mg^−1^) is shown. The Y axis is divided in two segments to better visualize the lower activities. In (**B**), the assays were performed with NADH and NADP (oxidized form) as a competitive inhibitor at indicated concentrations. Shown are relative activities, calculated as percent of the activity measured at the respective NADH concentration in the absence of NADP. Error bars represent standard deviation of technical triplicates. n.d., not detectable.

To investigate the molecular basis of these in vivo observations, we performed in vitro enzyme assays of AnGar1 variants. For this, we selected the wildtype enzyme, the single mutant producing the higher GalOA amount in the NADH background (R267L) and the double mutant K261M/R267L. Consistent with in vivo results, the single mutation only partly and the double mutation almost fully abolished the AnGar1 activity with NADPH as the cofactor (Fig. 6A). When the assay was performed with NADH, all three variants showed comparable activities, which were by an order of magnitude lower compared to those with NADPH of the wildtype enzyme (Fig. 6A). This demonstrates that (also wildtype) AnGar1 accepts NADH, albeit with a substantially lower preference compared to NADPH. However, when NADP was added to competitively inhibit NADH binding, only the mutants were able to efficiently reduce GalA using NADH (Fig. 6B). Whereas the double mutant was fully insensitive to NADP at both NADH concentrations tested, the single mutant showed a considerable sensitivity at the lower (160 µM) but not at the higher (800 µM) NADH concentration. This is consistent with its ability to use NADPH as a cofactor, in contrast to the double mutant (Figs. 5, 6A). Together, these data demonstrate that the change of the cofactor preference is due to a dramatically decreased affinity towards NADP(H), which is in accordance with the expectations based on the structure model. In the cellular context, the wildtype enzyme cannot use NADH supplied by Sor2 due to the presence of NADP (which accumulates in the absence of the enzyme that can re-reduce it, i.e. in the absence of YlSdr). Conversely, the mutated variants can bind NADH, since they are not sensitive to the presence of NADP. This notion also explains why AnGar1 mutants produce more GalOA in vivo in the Sor2 background compared to AnGaaA, which also accepts NADH, but with a by an order of magnitude lower affinity (reflected by the K_m_ value) compared to NADPH^18^.

To test the performance of yeast strains expressing mutated AnGar1 variants in a standardized batch processes, biotransformations were again studied in stirred-tank bioreactors. Here, the genes coding for the GalA reductase variants were integrated genomically as single copies for better stability^25^. As shown before in shake flasks, both singly (R267L) and doubly mutated (K261M/R267L) AnGar1 reductases could reduce GalA to GalOA. The *S. cerevisiae* strain with the singly substituted GalA reductase reached a final product concentration of 1.57 g L^−1^ GalOA (+ 27% compared to the results observed in shake flasks) and a final dry cell mass concentration of 3.7 g L^−1^. The strain expressing the doubly mutated enzyme produced 0.48 g L^−1^ GalOA (only 44% of the final product concentration reached in shake flasks) with a final dry cell mass concentration of 4.1 g L^−1^. It should be noted that the comparison of the results achieved in shake flasks and stirred-tank bioreactors is somewhat biased due to different expression levels, as multicopy plasmids were used in the shake flask experiments and genomically integrated single copy constructs in the stirred-tank bioreactor. Moreover, the aeration conditions and, consequently, the respiratory re-oxidation of NADH likely vary between the bioreactor and shake flask fermentations.

**Figure 7:**
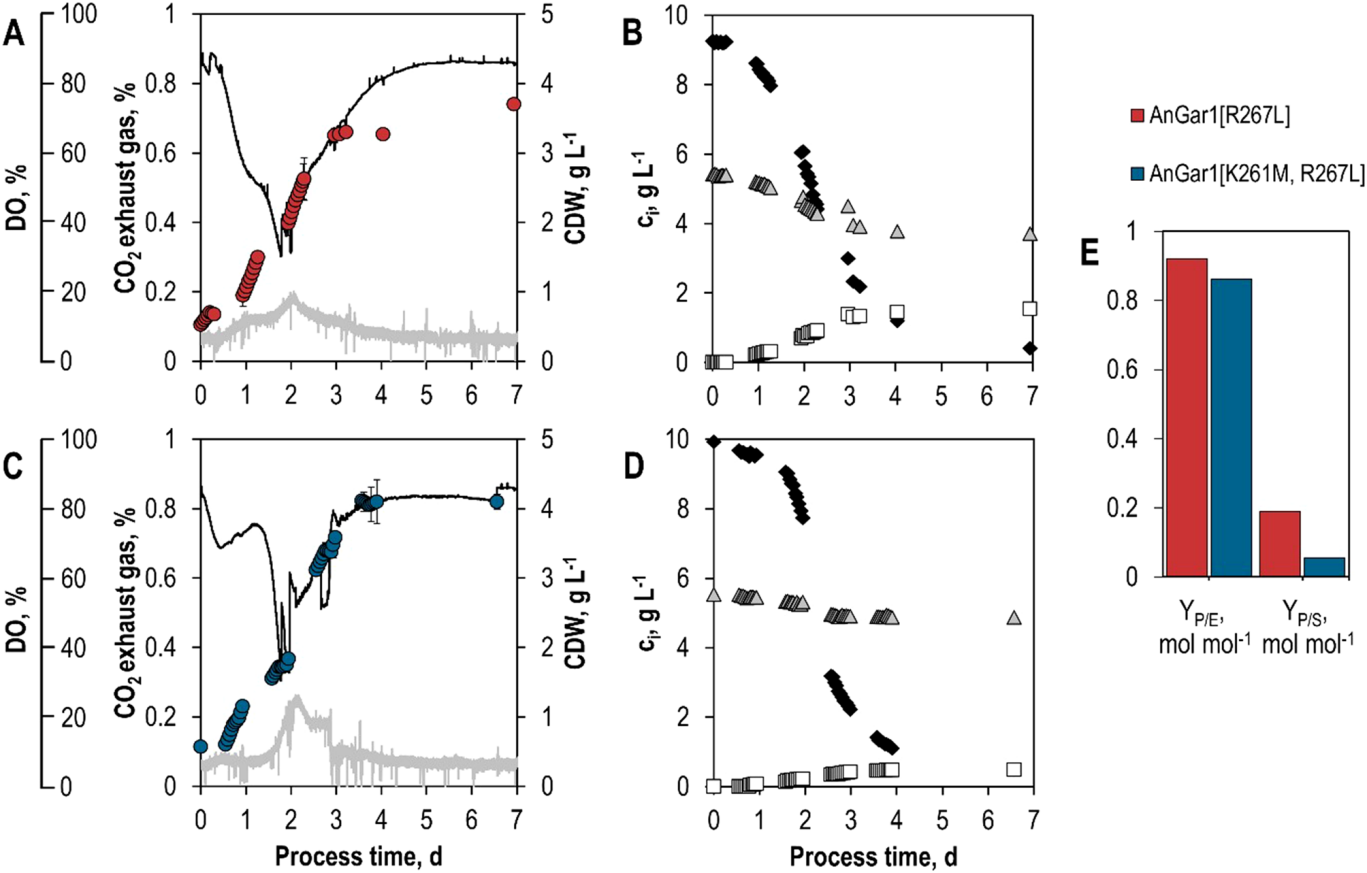
Aerobic batch fermentations of *S. cerevisiae* SiHY030 (Sor2, AnGar1[R267L]) (**A**, **B**) and SiHY032 (Sor2, AnGar1[K261M, R267L]) (**C**, **D**) in stirred-tank bioreactors. Cell dry weight concentrations (circles), online measured dissolved oxygen concentration (black line) and carbon dioxide concentration in the exhaust gas (grey line) as a function of the process time (**A**, **C**). Concentrations of sorbitol (black diamonds), GalA (grey triangles), and GalOA (white rectangles) as a function of the process time, as measured by HPLC in the fermentation broth (**B**, **D**). Final molar yield coefficients Y_P/E_ (GalOA/GalA) and Y_P/S_ (GalOA/sorbitol) in both batch processes (**E**).

The biotransformation of GalA was strongly growth-dependent. GalOA formation stagnated when the stationary phase was reached at approximately 72 h in both batch processes (Fig. 7A,C). The lower molar yields of GalOA produced per mol sorbitol (Y_P/S_) with NADH-dependent GalA reductase variants compared to the NADPH-dependent reductase may be caused by other NADH-consuming metabolic reactions in yeast. The difference in final product titers in batch processes making use of NADH-dependent GalA reductases might result from the reduced capability of the AnGar1 double mutant to utilize NADH and NADPH at the same time.

The estimated final product selectivity (Y_P/E_) of the biotransformation with the AnGar1 double mutant (0.86 mol GalOA per mol GalA) is not significantly lower compared to the single mutant (Y_P/E_ = 0.92 mol mol^−1^). The discrepancy, however, might come from the low final product concentrations of biotransformations with the double mutant being harder to distinguish analytically from the background noise in the UV-chromatogram of fermentation samples. The estimated integral carbon balance data in both batch processes could be closed (100%, considering an error range of ± 10%) (Supplementary Figures S5, S6).

### Co-fermentation of GalA and glucose using engineered GalA reductases

**Figure 8:**
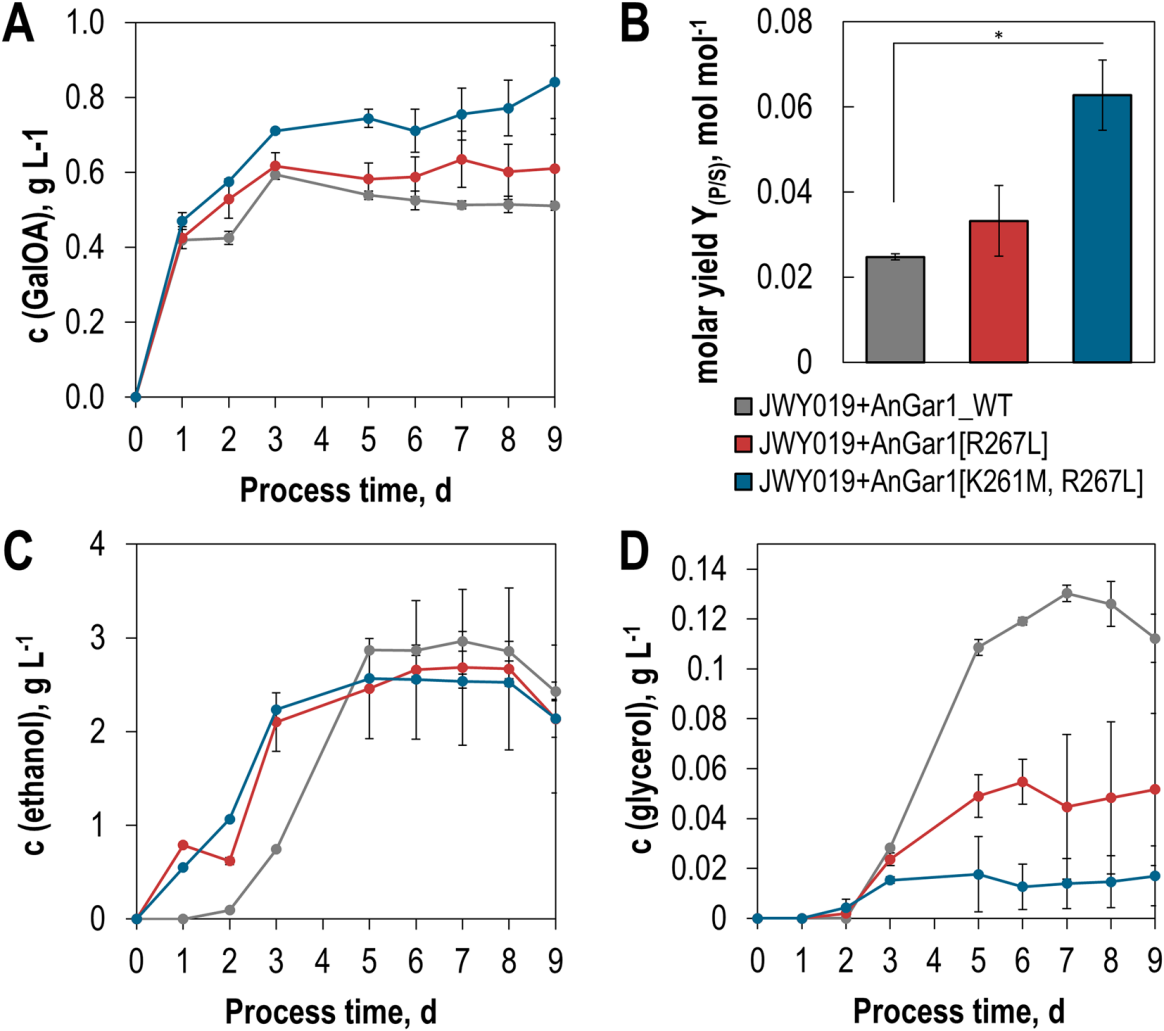
Performance of engineered GalA reductase variants in co-fermentations of GalA and glucose. Different GalA reductase variants (AnGar1_WT, AnGar1[R267L] and AnGar1[K261M/R267L] were integrated into the genome of the *adh1Δ gpd1Δ gpd2Δ* strain JWY019, yielding strains SiHY072, SiHY062 and SiHY063, respectively. The production of GalOA (**A**,**B**), ethanol (**C**) and glycerol (**D**) were measured in culture supernatants by HPLC analysis. In (**B**) the molar yields of GalOA (mol per mol consumed glucose) were calculated after 9 days of cultivation. The difference between the wildtype and the double mutant is statistically significant (*t*-test *P* < 0.005), whereas the difference between the wildtype and the single mutant is not (*P* > 0.05). The corresponding glucose consumption and growth curves are shown in Supplementary Fig. S7. Mean values and standard deviations of biological triplicates are shown. The same color code applies to all panels.

Sorbitol and GalA co-occur in fruits^26^, but some major sources of pectin, such as sugar beet pulp, do not contain a large amount of the sugar alcohol. Instead, this feedstock contains glucose, galactose, and arabinose as major carbohydrates^19^. All these substrates are funneled into glycolysis, which produces NADH in the GAPDH reaction. In *S. cerevisiae*, the predominant fraction of NADH is re-oxidized by the production of ethanol even under aerobic conditions in the presence of glucose due to the Crabtree effect^27,28^. Thus, glycolysis is redox-neutral, and “superfluous” NADH, resulting from the production of biomass, is re-oxidized through the production of glycerol^29^. To minimize the re-oxidation of NADH, we decided to test the performance of our engineered GalA reductase variants in the strain JWY019, which lacks the main alcohol dehydrogenase *ADH1* and the glycerol-phosphate-dehydrogenase genes *GPD1* and *GPD2*^30^. In this strain background, the engineered enzymes indeed achieve higher GalOA titers (Fig. 8A), and molar yields per mol consumed glucose (Fig. 8B).

With the K261M/R267L mutant, the yields were more than doubled compared to those observed with the wildtype enzyme. However, they are still below those obtained on sorbitol. This is obviously due to the fact that, despite the deletion of *ADH1*, *GPD1* and *GPD2*, NADH is still re-oxidized in JWY019 through the residual formation of ethanol (Fig. 8C) and glycerol (Fig. 8D). Strikingly, the production of glycerol is inversely correlated with the increased production of GalOA, supporting the conclusion that GalA acts as a redox sink for the glycolytic NADH. Our process optimization experiments (data not shown) and published results from others^9^ show that the production of GalOA under anaerobic conditions is not possible in *S. cerevisiae*. This observation can likely be attributed to the increased energy demand for transport of GalA, which requires proton symport^31^. Furthermore, it is possible that the export of GalOA occurs in an energy dependent manner, further compromising the energetic balance of the cell. Although further interventions into the NADH metabolism (e.g. the deletion of all alcohol dehydrogenase genes) are necessary to increase the yield of GalOA, our results demonstrate that NADH-dependent GalA reductases are useful to improve the co-fermentation of GalA with neutral sugars such as glucose or galactose.

## Conclusion

Here, we demonstrated that the supply of suitable redox cofactors is a critical engineering target to enable efficient reduction of GalA in *S. cerevisiae*. This can be achieved by feeding a co-substrate exhibiting a high reduction state, such as sorbitol (or other polyols). We show for the first time that AnGar1 has a GalA reductase activity. Using the sorbitol-based screening system and structure-guided mutagenesis, we developed AnGar1 variants that, to our knowledge, represent the first reported GalA reductases with a higher preference for NADH compared to NADPH. The altered cofactor-specificity enables the coupling of GalA reduction to glycolysis, resulting in higher yields of GalOA when glucose is used as a redox donor. Hence, the engineered AnGar1 should prove valuable for GalA utilization in pectin-rich hydrolysates, which contain neutral sugars such as glucose, galactose, or arabinose, all of which are funneled into glycolysis. Moreover, the NADH-dependent GalA reductases could facilitate the coupling of GalOA production to the oxidation of glycerol, an abundant waste product that could be supplemented to pectin-rich hydrolysates.

## Materials and methods

### Construction of expression cassettes and strains

The *Saccharomyces cerevisiae* endogenous open reading frames (ORFs) of *HXT13* (YEL069C) and *SOR2* (YDL246C) were PCR amplified using the primer pairs SiHP011-SiHP012 (*HXT13*) and SiHP015-SiHP016 (*SOR2*). The open reading frame encoding YlSdr^22^; UniProtKB—Q6CEE9) was amplified from *Yarrowia lipolytica* genomic DNA using the primer pair SiHP015-SiHP016 (primers are listed in Supplementary Table S1). Synthetic ORFs encoding the d-galacturonic acid reductases AnGaaA^18^; UniProtKB—A8DRH9), TrGar1^11^; UniProtKB—Q3ZFI7), and AnGar1 (UniProtKB—A2R7U3), as well as the d-galacturonic acid transporter AnGatA^10^; UniProtKB—A2R3H2) were codon optimized for expression in *S. cerevisiae* using the online tool JCat (https://www.jcat.de; Grote et al., 2005) and chemically synthesized at Thermo Fisher Scientific. The synthesized ORFs were cloned into pYTK001, the entry plasmid of the Golden Gate Cloning toolkit according to the published protocol^32^. The resulting plasmids, denoted as pGG3.x, are listed in Supplementary Table S2. Site directed mutagenesis for amino acid substitutions was performed on pGG3.6 (*AnGAR1*) and pGG3.7 (*TrGAR1*) using the primers listed in Supplementary Table S1. From the pGG3.x entry plasmids, integrative (SIHV046 and -47, Supplementary Table S2), or episomal (SiHV057-102, Supplementary Table S2) expression constructs were generated by combining the ORFs with modules of the Golden Gate toolkit as listed in Supplementary Table S2 according to the published procedure^32^.

Novel strains, SiHY001, SiHY002, SiHY003, SiHY004, SiHY007, and SiHY008 (Table 1), were constructed based on the parental strain EBY.VW4000^20^ by integrating expression cassettes from SiHV040, SiHV041, SiHV042, SiHV043, SiHV046, and SiHV047, which were previously digested with NotI. The strains SiHY062, SiHY063, and SiHY072, which are based on the parental strain JWY019^30^, were constructed via integration of the expression cassettes from plasmids SiHV136, SiHV137, and SiHV158 after NotI-digest. The cassettes were integrated into the *URA3* locus. Positive transformants were selected on G418 and PCR-verified. For testing the cofactor-dependent activity of different d-galacturonic acid reductase variants, the appropriate plasmids (SiHV057-102, Supplementary Table S2) were transformed into SiHY007 and SiHY008.

### Transformation of yeast cells

For yeast cell transformation, 50 mL YPD culture was inoculated with 1 mL of a YPD preculture and agitated at 200 rpm and 30 °C. The optical density was measured at 600 nm (OD_600_). When OD_600_ reached 0.8–1, the culture was pelleted at 3,000 × g for 3 min and washed with 25 mL sterile water. Cells equivalent to 5 OD_600_ units were pelleted at 5,000 × g for 1 min and used for one transformation. To this end, 240 μL 50% (w/w) polyethylene glycol, 36 μL 1 M lithium acetate, 10 μL ssDNA, and either 250 ng of plasmid-based or 5 µg of linear DNA in 64 μL water were added to the cells. The reaction set-up was mixed thoroughly and incubated at 42 °C for 20 min. Subsequently, the cells were pelleted at 5,000 × g for 30 s, resuspended in 500 μL YPD medium and spread on appropriate plates. Successfully transformed cells were expected to form colonies after 2–4 days of incubation at 30 °C.

### Cultivation of yeast cells in shake flasks

Colonies of strains transformed with plasmids for expression of different d-galacturonic acid reductase variants were scraped off for an overnight preculture in synthetic complete medium lacking uracil (SC-Ura) supplemented with 2% (w/v) maltose. Precultures of non-plasmid strains were started from a single colony in synthetic complete medium with all essential medium compounds supplemented. The main culture was grown in a 300 mL shake flask in 50 mL SC medium^33^ lacking uracil and supplemented with 0.5% (w/v) d-galacturonic acid and 1% (w/v) sorbitol or 2% (w/v) glucose, respectively, at 30 °C and shaking at 200 rpm. The medium was buffered with 100 mM potassium phosphate, pH 6.3. The growth was monitored through OD_600_-measurement, and samples were withdrawn for HPLC-analysis.

#### HPLC analysis

The samples were treated with 5-sulfosalicylic acid to a final concentration of 5% (w/v). The analysis was done with an Ultimate 3000 HPLC system (Thermo Fisher Scientific) equipped with a NucleoGel Sugar 810 H (Macherey and Nagel) column. The column temperature was set to 30 °C, and the eluent (5 mM H_2_SO_4_) flow rate was 0.4 mL/min under isocratic conditions. The signal was recorded using a refractive index detector (Shodex RI-101, Shoko Scientific Co.).

#### Protein extraction and enzyme assays

CEN.PK2-1C cells transformed with AnGar1 plasmids (SiHV079, SiHV101 and SiHV102) or with the empty plasmid as a control were grown in 50 ml SC-Ura media containing 2% (w/v) glucose until an OD_600_ = 2.0–2.5. Subsequently, cells were harvested by centrifugation, washed and stored at -80 °C until further processing. After thawing on ice, the cells were mechanically disrupted in 10 mM potassium phosphate buffer (pH 7.2) by shaking (10 min at 4 °C) with glass beads (0.45 mm diameter) using a Vibrax cell disruptor (Janke & Kunkel, Staufen, Germany) and the cell debris was subsequently removed by centrifugation (15,000 × g, 5 min, 4 °C). Protein concentration of clear crude extracts was determined by the Bradford method, using bovine serum albumin as a standard. Enzyme assays were performed basically as described previously^18^. In detail, the reaction mixtures contained (in 200 µl) 10 mM potassium phosphate buffer (pH 7.2), 100 mM GalA, 160 or 800 µM NADPH or NADH and NADP as a competitive inhibitor, where indicated. The reaction was started by adding 10 µl of the substrate solution. The oxidation of NAD(P)H during 10 min was recorded by measuring the change of the absorbance at 340 nm. The specific activities (expressed as mili Units, mU per mg protein) were calculated by dividing the slope measured at 340 nm by the reaction time and protein amount in the reaction mixture.

### Batch processes in stirred-tank bioreactors and offline analytics

Seed cultures for inoculation of the stirred-tank bioreactor were prepared in 1000 mL shake flasks without baffles with 100 mL of the same SC medium used for batch processes in stirred-tank bioreactors containing 1.7 g L^−1^ BD™ Difco™ yeast nitrogen base without amino acids and ammonium sulfate (Fisher Scientific GmbH, Schwerte, Germany), amino acid mix (0.056 g L^−1^ adenine, 0.192 g L^−1^ arginine, 0.192 g L^−1^ methionine, 0.072 g L^−1^ tyrosine, 0.288 g L^−1^ isoleucine, 0.323 g L^−1^ lysine*H_2_O, 0.240 g L^−1^ phenylalanine, 0.288 g L^−1^ valine, 0.288 g L^−1^ threonine, 0.096 g L^−1^ uracil, 0.096 g L^−1^ histidine, 0.095 g L^−1^ tryptophan, 0.288 g L^−1^ leucine), 10 g L^−1^ ammonium sulfate, 10 g L^−1^ sorbitol, and 5 g L^−1^ d-galacturonic acid. Shake flasks were inoculated with 500 µL glycerol stocks of *S. cerevisiae* mutants. Inoculum for the stirred-tank bioreactors was prepared at 30 °C and 180 rpm (Multitron, Infors HT, Bottmingen, Switzerland). After 72 h, the adapted cells were harvested by centrifugation and washed twice with sterile phosphate buffered saline (8 g L^−1^ NaCl, 0.2 g L^−1^ KCl, 1.44 g L^−1^ Na_2_HPO_4_, 0.24 g L^−1^ KH_2_PO_4_, pH 7.4) at 3,000 × g within 10 min at 4 °C (Rotixa 50 RS, Andreas Hettich GmbH& Co.KG, Tuttlingen, Germany). The cell pellet was re-suspended in 10 mL sterile SC medium, and a sterile single-use syringe (BD Discardit II, Becton Dickinson, Franklin Lakes, USA) and sterile cannula (Sterican 0.8 × 120 mm, B. Braun, Melsungen, Germany) were used for inoculation of the bioreactor with an amount equivalent to 0.25 g L^−1^ cell dry weight (CDW).

Batch processes were performed with a lab-scale stirred-tank bioreactor on a 3.6 L scale (Labfors 2, Infors HT, Bottmingen, Switzerland) equipped with 4 baffles, 2 six-blade Rushton turbines and a working volume of 1.2 L. Temperature, pressure, dissolved oxygen (DO) concentration (air saturation), pH, and off-gas flow rate were monitored with the process control software IRIS 5 (Infors HT, Bottmingen, Switzerland). Gas concentrations of O_2_ and CO_2_ were monitored online with a gas-analyzer (EasyLine, ABB, Zurich, Switzerland). The pH was controlled at pH 5.0 with 1 M KOH and 0.5 M sulfuric acid. Two-point DO calibration was performed after autoclaving and prior to inoculation at 2 vvm and 600 rpm by stripping the medium with nitrogen gas until 0% air saturation and afterwards with air until 100% air saturation was achieved. DO concentration below 100% air saturation after inoculation is caused by the initially reduced stirrer speed of 200 rpm. Aeration, temperature, and pressure were kept constant at 0.5 vvm, 30 °C, and 1 bar, respectively. DO concentration was kept above 30% air saturation by automatic increase of the stirrer speed from initially 200 rpm to 600 rpm at the maximum.

Cell densities were determined by measuring the optical density at a wavelength of 600 nm (OD_600_) in 10-mm cuvettes using a single beam photometer (Genesys 10S UV–VIS, Thermo Scientific, Neuss, Germany). For determination of cell dry weight (CDW) concentrations, 2 mL cell suspension was withdrawn and centrifuged for 10 min at 13,000 × g in pre-dried (24 h, 80 °C) and pre-weighted reaction tubes. After discarding the supernatant, the cell pellet was dried for another 24 h at 80 °C, and cell dry weight was determined gravimetrically as triplicates.

High performance liquid chromatography (HPLC) analysis was used for metabolite detection in the fermentation broth. Samples were centrifuged for 10 min at 13,000 × g, and the supernatant was filtered before analysis (0.22 μm pore size, Chromafil RC20/15 MS, Macherey–Nagel GmbH & Co.KG, Düren, Germany). An Aminex HPX-87H column (Biorad, Munich, Germany) was used for separation of substrates and products applying HPLC (Agilent 1100, Agilent Technologies Inc., Santa Clara, USA) equipped with an RI detector (Agilent 1200, Agilent Technologies Inc., Santa Clara, USA) and a thermostat (Mistral, Spark Holland, VE Emmen, the Netherlands). Twenty microliters of samples (or standards) was injected, and separation was performed at a constant column temperature of 60 °C, at a flow rate of 0.5 mL min^−1^ with 5 mM sulfuric acid as the mobile phase.

#### Modeling of AnGar1 and TnGar1

The homology models of AnGar1 and TrGar1 were generated with the ‘Homology Model’ function of the program package Molecular Operating Environment (MOE; Chemical Computing Group, https://www.chemcomp.com/), using as a template the crystal structure of the NADPH-dependent aldehyde reductase AKR1A1 from *Sus scrofa* (PDB ID 1HQT). The amino acid sequence identity and similarity between AKR1A1 and AnGar1 (or TnGar1) are 37% and 59%, respectively. The homology models generated were scored with GB/VI. The mutation residue scan and resulting protein stability and ligand affinity parameters were performed in MOE Protein Designing function with the Forcefields Amber10 and EHT.

## Supplementary information

Supplementary Information.

## Data Availability

The authors will make available all data (underlying the described findings) without restriction upon request.
