# Supplementary material for: Engineering cofactor supply and NADH-dependent d-galacturonic acid reductases for redox-balanced production of l-galactonate in Saccharomyces cerevisiae
Source: Sci Rep. 2020 Nov 4;10:19021. doi: 10.1038/s41598-020-75926-5 (PMC7642425; doi:10.1038/s41598-020-75926-5)
Supplement: Supplementary file 1 — Supplementary Information. [file 41598_2020_75926_MOESM1_ESM.pdf]

## SUPPLEMENTARY INFORMATION

# Engineering cofactor supply and NADH-dependent D-galacturonic acid reductases for redox-balanced production of L-galactonate in *Saccharomyces cerevisiae*

Simon Harth[1], Jacqueline Wagner[2], Tamina Sens[1], Jun-yong Choe[3,4], J. Philipp Benz[5], Dirk Weuster-Botz[2] & Mislav Oreb[1*]

[1]Goethe University Frankfurt, Faculty of Biological Sciences, Institute of Molecular Biosciences, Max-von-Laue Straße 9, 60438 Frankfurt am Main, Germany

[2]Technical University of Munich, Institute of Biochemical Engineering, Boltzmannstr. 15, 85748 Garching, Germany

[3]East Carolina University, Department of Chemistry, East Carolina Diabetes and Obesity Institute, 115 Heart Drive, Greenville, NC 27834, USA

[4]Rosalind Franklin University of Medicine and Science, Department of Biochemistry and Molecular Biology, 3333 Green Bay Road, North Chicago, IL 60064, USA.

[5]Technical University of Munich, TUM School of Life Sciences Weihenstephan, Holzforschung München, Hans-Carl-von-Carlowitz-Platz 2, 85354 Freising, Germany

*corresponding author:
Dr. Mislav Oreb
Institute of Molecular Biosciences
Max-von-Laue Straße 9
60438 Frankfurt
Germany

| | |
|---|---|
| Telephone | +49 (0)69 798 29331 |
| Telefax | +49 (0)69 798 29527 |
| E-Mail | m.oreb@bio.uni-frankfurt.de |

## Supplementary Table S1: List of oligonucleotides used in the study

| Primer name | Target | Sequence |
|---|---|---|
| **SiHP011** | HXT13_fw | CGTCTCGTCGGTCTCATATGTCTAGTGCGCAATCCTCTA |
| **SiHP012** | HXT13_rev | CGTCTCAGGTCGGTCTCAGGATTCAATCAGAATTCTTTGAGAACTTC |
| **SiHP013** | YlSDR_fw | CGTCTCGTCGGTCTCATATGCCTGCACCAGCAAC |
| **SiHP014** | YlSDR_rev | CGTCTCAGGTCGGTCTCAGGATTCAAGGACAACAGTAGCCGC |
| **SiHP015** | SOR2_fw | CGTCTCGTCGGTCTCATATGTCTCAAAATAGTAACCCTGCAGT |
| **SiHP016** | SOR2_rev | CGTCTCAGGTCGGTCTCAGGATTCATTCAGGACCAAAGATAATAGTCT T |
| **SiHP046** | TrGar1: K254M_fw | GGTTCTACTGTTTTGGCTATGTCTGTTACTCCAGCT |
| **SiHP047** | TrGar1: K254M_rev | GCTGGAGTAACAGACATAGCCAAAACAGTAGAACCTCTG |
| **SiHP048** | TrGar1: R260L_fw | TACTCCAGCTCTGATCAAGGCTAACTTGGAAATCGTTGACTTGGA |
| **SiHP049** | TrGar1: R260L_rev | AGTTAGCCTTGATCAGAGCTGGAGTAACAGACTTAGCCAAAACAGTAG AACC |
| **SiHP050** | TrGar1: K254M, R260L_rev | AGTTAGCCTTGATCAGAGCTGGAGTAACAGACATAGCCAAAACAGTAG AACCTCTGTTAACGTGG |
| **SiHP051** | AnGar1: K261M_fw | CTGTTTTGGCTATGTCTGTTAACCCATCTAGAATCGAAGG |
| **SiHP052** | AnGar1: K261M_rev | GATGGGTTAACAGACATAGCCAAAACAGAAGAACCTCTAGAGATGTGC |
| **SiHP053** | AnGar1: R267L_fw | GTCTGTTAACCCATCTCTGATCGAAGGTAACAGAAACTTGGTTGCTTTG G |
| **SiHP054** | AnGar1: R267L_rev | CCAAGTTTCTGTTACCTTCGATCAGAGATGGGTTAACAGACTTAGCCAA AACAGAAGAAC |
| **SiHP055** | AnGar1: K261M, R267L_rev | CCAAGTTTCTGTTACCTTCGATCAGAGATGGGTTAACAGACATAGCCAA AACAGAAGAACCTCTAGAGATGTGC |

## Supplementary Table S2: List of plasmids used in this study

| Plasmid name | Content | Source |
|---|---|---|
| **pGG3.2** | *pYTK001-HXT13* | this study |
| **pGG3.3** | *pYTK001-YlSDR* | this study |
| **pGG3.4** | *pYTK001-SOR2* | this study |
| **pGG3.6** | *pYTK001-AnGAR1* | this study |
| **pGG3.6 K261M** | *pYTK001-AnGAR1 [K261M]* | this study |
| **pGG3.6 R267L** | *pYTK001-AnGAR1 [R267L]* | this study |
| **pGG3.6 K261M, R267L** | *pYTK001-AnGAR1 [K261M, R267L]* | this study |
| **pGG3.7** | *pYTK001-TrGAR1* | this study |
| **pGG3.7 K254M** | *pYTK001-TrGAR1 [K254M]* | this study |
| **pGG3.7 R260L** | *pYTK001-TrGAR1 [R260L]* | this study |
| **pGG3.7 K254M, R260L** | *pYTK001-TrGAR1 [K254M, R260L]* | this study |
| **pGG3.9** | *pYTK001-AnGATA* | this study |
| **pGG3.18** | *pYTK001-AnGAAA* | this study |
| **SiHV005** | *Empty expression backbone pURA3-URA3-tURA3, 2µ, KanR-ColE1* | this study |
| **SiHV033** | *-URA3 5'Hom-ConLS'-GFP-dropout-ConRE'-KanMX-URA 3'Hom-KanR-ColE1 URA3 integration plasmid, with KanMX dominant marker* | this study |
| **SiHV040** | *URA3_5'-pCCW12-AnGATA-tPGK1-pPGK1-AnGAR1-tENO1-pTDH3-HXT13-tSSA1-pTEF2-YlSDR-tADH1- pAgTEF-KanMX-tAgTEF-URA3_3'-KanR-ColE1* | this study |
| **SiHV041** | *URA3_5'-pCCW12-AnGATA-tPGK1-pPGK1-TrGAR1-tENO1-pTDH3-HXT13-tSSA1-pTEF2-YlSDR-tADH1- pAgTEF-KanMX-tAgTEF-URA3_3'-KanR-ColE1* | this study |
| **SiHV042** | *URA3_5'-pCCW12-AnGATA-tPGK1-pPGK1-AnGAR1-tENO1-pTDH3-HXT13-tSSA1-pTEF2-SOR2-tADH1- pAgTEF-KanMX-tAgTEF-URA3_3'-KanR-ColE1* | this study |
| **SiHV043** | *URA3_5'-pCCW12-AnGATA-tPGK1-pPGK1-TrGAR1-tENO1-pTDH3-HXT13-tSSA1-pTEF2-SOR2-tADH1- pAgTEF-KanMX-tAgTEF-URA3_3'-KanR-ColE1* | this study |
| **SiHV046** | *URA3_5'-pCCW12-AnGATA-tPGK1-pTDH3-HXT13-tSSA1-pTEF2-YlSDR-tADH1-pAgTEF-KanMX-tAgTEF-URA3_3'- KanR-ColE1* | this study |
| **SiHV047** | *URA3_5'-pCCW12-AnGATA-tPGK1-pTDH3-HXT13-tSSA1-pTEF2-SOR2-tADH1-pAgTEF-KanMX-tAgTEF-URA3_3'- KanR-ColE1* | this study |
| **SiHV057** | *pPGK1-AnGAAA-tENO1, pURA3-URA3-tURA3, 2µ, KanR-ColE1* | this study |
| **SiHV058** | *pPGK1-TrGAR1 [K254M]-tENO1, pURA3-URA3-tURA3, 2µ, KanR-ColE1* | this study |
| **SiHV059** | *pPGK1-TrGAR1 [R260L]-tENO1, pURA3-URA3-tURA3, 2µ, KanR-ColE1* | this study |
| **SiHV060** | *pPGK1-TrGAR1 [K254M, R260L]-tENO1, pURA3-URA3-tURA3, 2µ, KanR-ColE1* | this study |
| **SiHV074** | *pPGK1-TrGAR1-tENO1, pURA3-URA3-tURA3, 2µ, KanR-ColE1* | this study |
| **SiHV079** | *pPGK1-AnGAR1-tENO1, pURA3-URA3-tURA3, 2µ, KanR-ColE1* | this study |
| **SiHV100** | *pPGK1-AnGAR1 [K261M]-tENO1, pURA3-URA3-tURA3, 2µ, KanR-ColE1* | this study |
| **SiHV101** | *pPGK1-AnGAR1 [R267L]-tENO1, pURA3-URA3-tURA3, 2µ, KanR-ColE1* | this study |
| **SiHV102** | *pPGK1-AnGAR1 [K261M, R267L]-tENO1, pURA3-URA3-tURA3, 2µ, KanR-ColE1* | this study |
| **SiHV115** | *pTEF1-AnGAR1 [K261M, R267L]-tTDH1, URA3, 2µ, KanR-ColE1* | this study |

| | | |
|---|---|---|
| **SiHV127** | *pTEF1-AnGAR1 [R267L]-tTDH1, URA3, 2µ, KanR-ColE1* | this study |
| **SiHV128** | *URA3_5'-pCCW12-AnGATA-tPGK1-pPGK1-AnGAR1 [K261M]-tENO1-pTDH3-HXT13-tSSA1-pTEF2-SOR2-tADH1- pAgTEF-KanMX-tAgTEF-URA3_3'- KanR-ColE1* | this study |
| **SiHV129** | *URA3_5'-pCCW12-AnGATA-tPGK1-pPGK1-AnGAR1 [K261M, R267L]-tENO1-pTDH3-HXT13-tSSA1-pTEF2-SOR2-tADH1- pAgTEF-KanMX-tAgTEF-URA3_3'- KanR-ColE1* | this study |
| **SiHV136** | *URA3_5'-pCCW12-AnGATA-tPGK1-pPGK1-AnGAR1[R267L]-tENO1-pAgTEF-KanMX-tAgTEF-URA3_3'- KanR-ColE1* | this study |
| **SiHV137** | *URA3_5'-pCCW12-AnGATA-tPGK1-pPGK1-AnGAR1 [R267L, K261M]-tENO1- pAgTEF-KanMX-tAgTEF-URA3_3'- KanR-ColE1* | this study |
| **SiHV158** | *URA3_5'-pCCW12-AnGATA-tPGK1-pPGK1-AnGAR1-tENO1- pAgTEF-KanMX-tAgTEF-URA3_3'- KanR-ColE1* | this study |

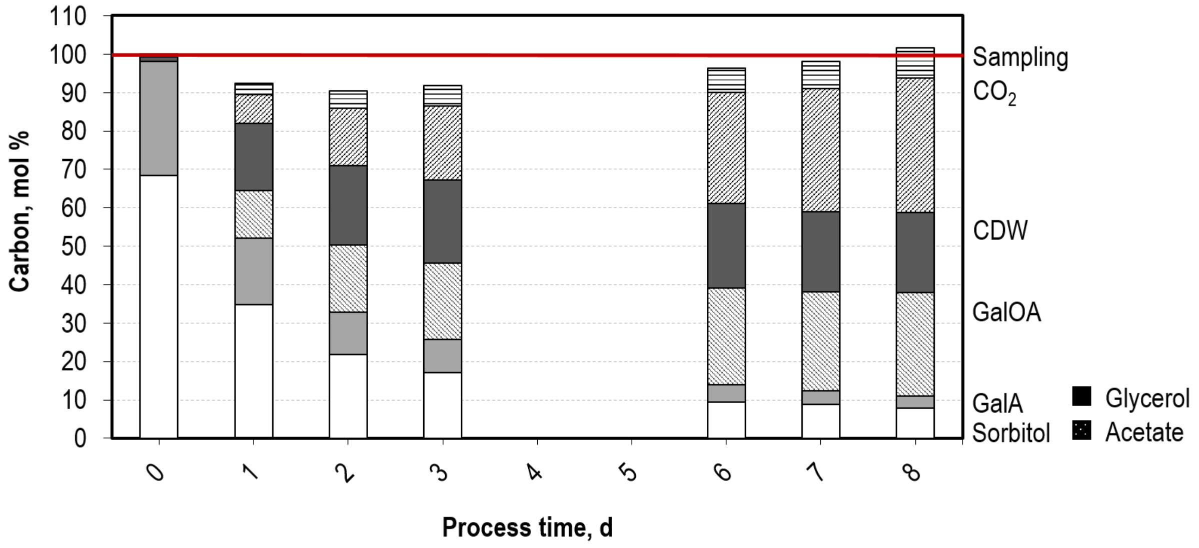


**Supplementary Figure S3. Integral carbon balance of aerobic batch fermentation of *S. cerevisiae* SiHY001 in a stirred-tank bioreactor.** The bars depict the amount of carbon (mol %) of each measured component at the sample time in respect to the total sum of carbon in the system. For estimation of carbon content in cell dry weight (CDW), the biomass composition was assumed to be $CH_{1,82}O_{0,58}N_{0,16}$ (Villadsen et al., 2011). Concentration of carbon dioxide (mol $h^{-1}$) was integrated over the process time using MatLab's trapz function (MATLAB, 2017, 9.3.0.713579 (R2017b), Natick, Massachusetts: The MathWorks Inc.). "Sampling" depicts loss of carbon due to sampling. Glycerol and acetate could only be detected in small amounts in the first 48 h of the process.

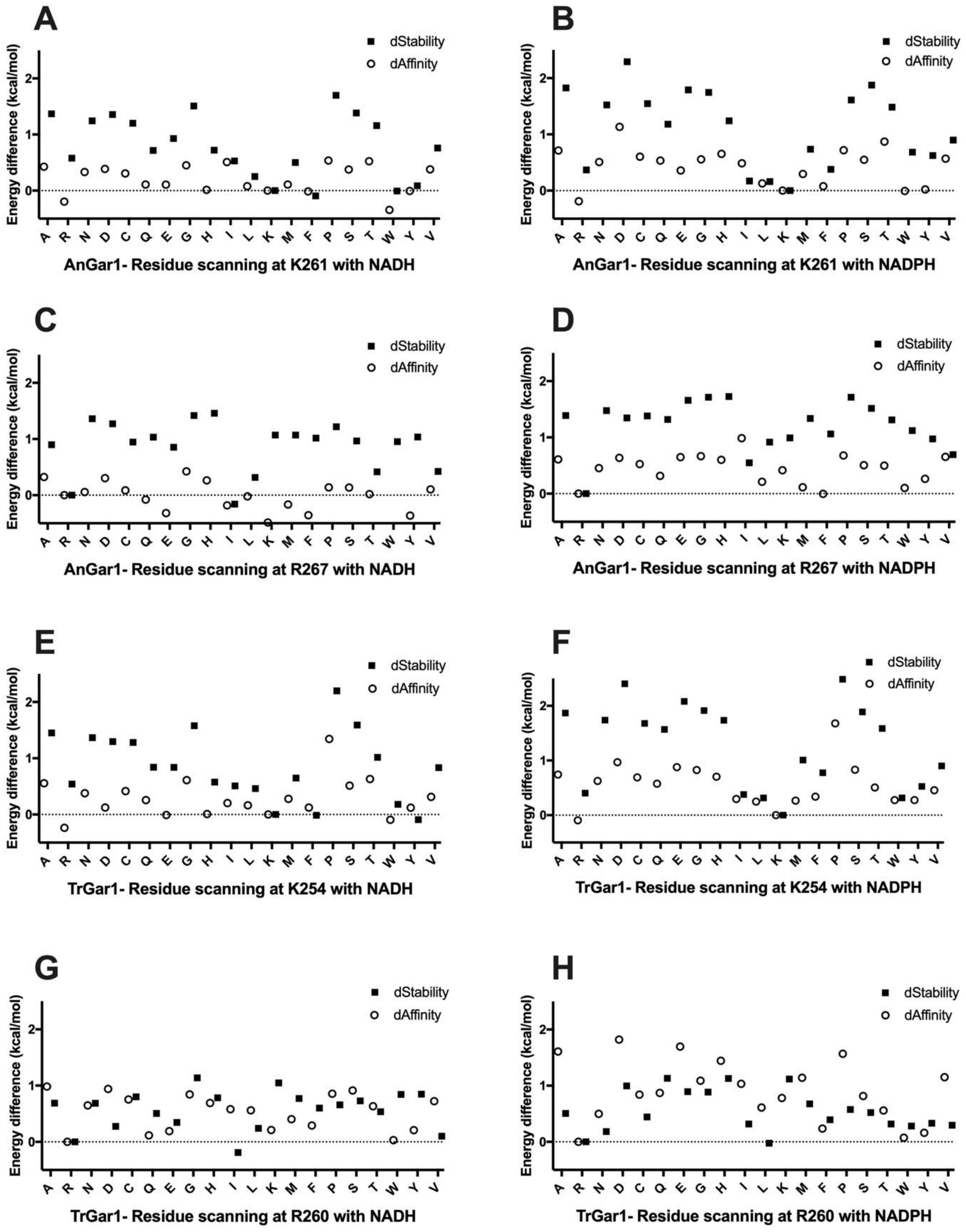


**Supplementary Figure S4. Graphical representation of the differences in protein stability (dStability) and ligand affinity (dAffinity) for mutants of the lysine and arginine residues interacting with the phosphoryl group of NADPH in TrGar1 and AnGar1.** Differences in the protein stability (black squares) and ligand affinity (empty circles) for various substitutions of K261 (A, B) and R267 (C, D) in AnGar1 or K254 (E, F) and R260 (G, H) in TrGar1, with NADH or NADPH, were calculated in MOE (Molecular Operating Environment, Chemical Computing Group, https://www.chemcomp.com/).

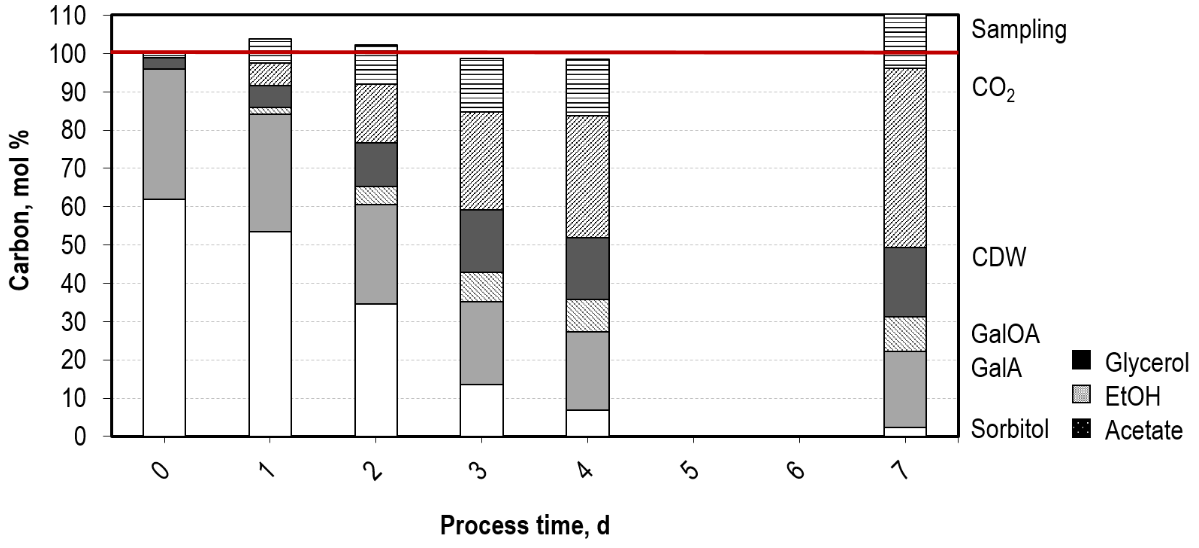


**Supplementary Figure S5. Integral carbon balance of aerobic batch fermentation of *S. cerevisiae* SiHY030 (Sor2, AnGar1[R267L]) in a stirred-tank bioreactor.** The bars depict the amount of carbon (mol %) of each measured component at the sample time in respect to the total sum of carbon in the system. For estimation of carbon content in cell dry weight (CDW), the biomass composition was assumed to be $CH_{1,82}O_{0,58}N_{0,16}$ (Villadsen et al., 2011). Concentration of carbon dioxide (mol $h^{-1}$) was integrated over the process time using MatLab's trapz function (MATLAB, 2017, 9.3.0.713579 (R2017b), Natick, Massachusetts: The MathWorks Inc.). "Sampling" depicts loss of carbon due to sampling. Glycerol, ethanol, and acetate could only be detected in small amounts in the first 48 h of the process.

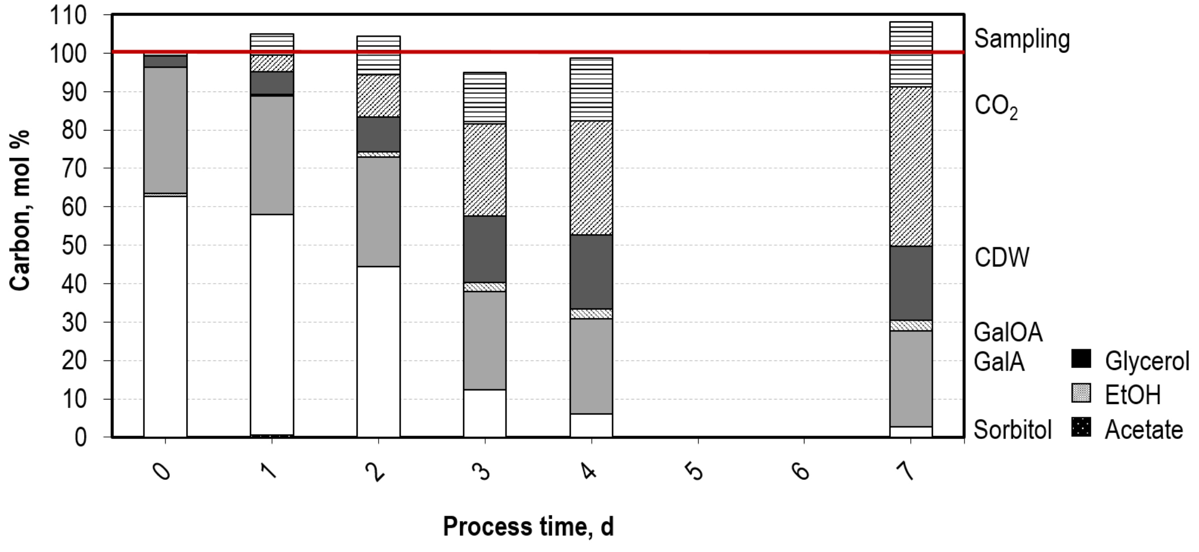


**Supplementary Figure S6. Integral carbon balance of aerobic batch fermentation of *S. cerevisiae* SiHY032 (Sor2, AnGar1[K261M, R267L]) in a stirred-tank bioreactor.** The bars depict the amount of carbon (mol %) of each measured component at the sample time in respect to the total sum of carbon in the system. For estimation of carbon content in cell dry weight (CDW), the biomass composition was assumed to be $CH_{1,82}O_{0,58}N_{0,16}$ (Villadsen et al., 2011). Concentration of carbon dioxide (mol $h^{-1}$) was integrated over the process time using MatLab's trapz function (MATLAB, 2017, 9.3.0.713579 (R2017b), Natick, Massachusetts: The MathWorks Inc.). "Sampling" depicts loss of carbon due to sampling. Glycerol, ethanol, and acetate could only be detected in small amounts in the first 48 h of the process.

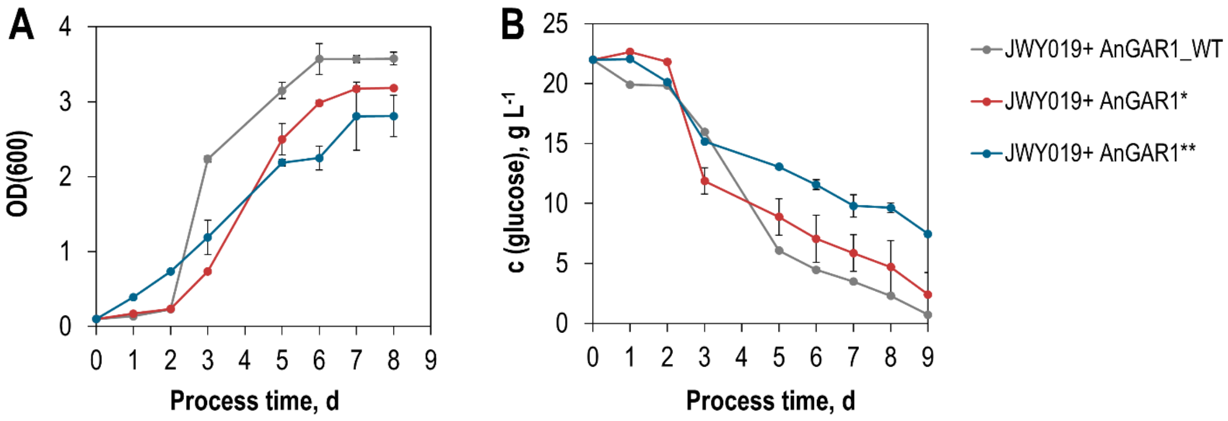


**Supplemetary Figure S7. Performance of engineered GalA reductase variants in co-fermentations of GalA and glucose.** Different GalA reductase variants (AnGar1_WT, AnGar1[R267L] and AnGar1[K261M/R267L] were integrated into the genome of the *adh1Δ gpd1Δ gpd2Δ* strain JWY019, yielding strains SiHY072, SiHY062 and SiHY063, respectively. The $OD_{600}$ (A) and the glucose concentration in the supernatant (B) were monitored over the cultivation time of 9 days via photometry and HPLC analysis, respectively. Mean values and standard deviations of biological triplicates are shown. The same color code applies to both panels.

## Supplementary Reference

Villadsen, J., Nielsen, J., & Lidén, G. (2011). Elemental and Redox Balances (Chapter 3, Table 3.2). In *Bioreaction Engineering Principles*. Springer US. https://doi.org/10.1007/978-1-4419-9688-6
